# Cascades From Early Adolescent Impulsivity to Late Adolescent Antisocial Personality Disorder and Alcohol Use Disorder

**DOI:** 10.1016/j.jadohealth.2022.06.007

**Published:** 2022-08-05

**Authors:** Ivy N. Defoe, Atika Khurana, Laura M. Betancourt, Hallam Hurt, Daniel Romer

**Affiliations:** a Forensic Child and Youth Care Sciences, University of Amsterdam, Amsterdam, The Netherlands; b Counseling Psychology and Human Services Department, University of Oregon, Eugene, Oregon; c Division of Neonatology, The Children’s Hospital of Philadelphia, Philadelphia, Pennsylvania; d Annenberg Public Policy Center, The University of Pennsylvania, Philadelphia, Pennsylvania

**Keywords:** Impulsivity, Alcohol use, Antisocial behavior, Adolescence, Developmental cascade models

## Abstract

**Purpose:**

The behavioral disinhibition model (BDM) posits that a liability toward impulsivity evident by early adolescence underlies the coemergence of antisocial behavior and alcohol use (i.e., problem behaviors) in early-adolescence to mid-adolescence, but that the subsequent development of these problem behaviors (rather than impulsivity itself) predicts the emergence of antisocial personality disorder (APD) and alcohol use disorder (AUD) in late adolescence. The present study was designed to test these predictions of the BDM from early to late adolescence.

**Methods:**

We used five-year longitudinal self-report data from the Philadelphia Trajectory Study that was collected from 2006–2012. Mediational analyses were performed using the Random Intercept Cross-lagged Panel Model, which enables the detection of within-person predictions of changes in problem behaviors during adolescence. The sample was ethnically and socioeconomically diverse, including 364 urban US community youth (at baseline: *M*_age_ = 13.51(.95); 49.1% female).

**Results:**

Consistent with the BDM, mediational analyses revealed that changes in early adolescent impulsivity predicted late adolescent APD and AUD criteria, mediated by changes in mid-adolescent alcohol use and conduct problems.

**Discussion:**

Interventions targeting impulsivity in early adolescence could potentially halt the cascading chain of events leading to both late adolescent APD and AUD by decelerating growth in antisocial behavior and alcohol use during early-adolescence to mid-adolescence. From mid-adolescence to late-adolescence, the consequences of early impulsivity, especially involvement in antisocial behaviors, become a more relevant predictor of both APD and AUD rather than impulsivity itself.

The heightened co-occurrence of antisocial behavior and substance use during adolescence can predict subsequent psychopathology (e.g., antisocial personality disorder [APD] and substance use disorder [SUD] [1]) and their co-occurrence is associated with a poorer prognosis [2–4]. For example, recent meta-analyses have revealed that the comorbidity between alcohol use disorder (AUD) and APD is as high as 76.6% [5] and that such comorbidity predicts treatment dropout [6]. Differences in impulsivity have long been acknowledged as important predictors of adolescent antisocial behavior and alcohol use [7,8] and subsequent disorders. As per the behavioral disinhibition model (BDM), impulsivity, evident by early adolescence, underlies the co-emergence of antisocial behavior and substance use in early and mid-adolescence [1]. By late adolescence, these problem behaviors predict the subsequent development of APD and SUDs, such as AUD [1,9].

The BDM hypothesizes that the cascading effects of impulsivity on early to mid-adolescent antisocial behavior and substance use [10] contribute to these problem behaviors becoming more direct predictors of psychopathology in late adolescence rather than impulsivity itself [1,9]. In addition, it has long been acknowledged that antisocial behavior (such as stealing and fighting) and substance use co-occur, with antisocial behavior typically emerging first [11]. As such, early impulsivity may continue to predict later alcohol use and AUD, through the cascading chains of mid to late adolescent antisocial behavior, but not directly [1,9]. Indeed, most studies suggest that adolescent impulsivity becomes less predictive of problem behaviors after mid-adolescence or late-adolescence [12–15].

While there is evidence to support the BDM [16], this research has focused on individual differences in impulsivity as predictors of subsequent individual differences in psychopathology. However, such tests of the theory do not directly inform us whether changes in impulsivity during adolescence only predict subsequent outcomes such as AUD and APD via behavioral tendencies such as alcohol use and antisocial behavior that emerge from impulsivity. That is, prior tests of the BDM have not reliably demonstrated whether impulsivity only continues to predict AUD and APD as mediated by the behaviors (i.e., alcohol use and antisocial behavior) that lead to those conditions. The present study was designed to test this prediction. If this prediction of the BDM were to hold, it would suggest that intervening on impulsivity will only provide benefit if done before its sequelae have emerged.

A relevant study by Elkins et al. [16] that showed support of BDM, used a twin-sample and 3-wave longitudinal design and reported that conduct disorder symptoms at age 11 predicted substance use at age 14 and SUD at 18, controlling for attention deficit hyperactivity disorder symptoms. However, the odds ratios for conduct disorder as a predictor of SUD were at least 1.5 times larger than the odds ratios of attention deficit hyperactivity disorder symptoms. These findings provide support for BDM but also suggest that besides impulsivity, conduct problems can begin to play a bigger role in predicting substance use especially later in adolescence. One explanation is that although impulsivity may decline in high-risk adolescents after its initial increase [17], the youth in that high-risk trajectory still show increased AUD later on [17]. Considering the findings of Elkins et al. [16], the abovementioned finding [17] emerged perhaps due to the sequelae of impulsivity via conduct problems leading to late AUD and APD. But this explanation is yet to be tested.

The present study uses repeated measurements of impulsivity, antisocial behavior, and alcohol use from early to late adolescence to test this cascading hypothesis. Namely, we simultaneously investigated (a) whether within-person changes in repeatedly measured impulsivity predict subsequent within-person changes in adolescent alcohol use and antisocial behavior (cf. [18]) and (b) whether those problem behaviors predict subsequent psychopathology (AUD and APD) in late adolescence rather than impulsivity itself (Figure 1). The longitudinal design is an advantage of the present study, because despite the support for the BDM, in many studies impulsivity is assumed to be stable and it is either measured in childhood or early adulthood [19]. In fact, in a recent meta-analysis [20] on self-control and problem behaviors, none of the included longitudinal adolescent studies had multiple assessments of impulsivity across the entire adolescent period. This leaves the potential role of impulsivity as a predictor of subsequent problem behavior across the adolescent period unexamined.

## Methods

### Participants

The community sample of adolescents in the present study took part in the Philadelphia Trajectory Study [21], which was approved by the Institutional Review Board of Children’s Hospital of Philadelphia. In this 6-wave study, participants between ages 10–12 years at baseline were tested annually from 2004 to 2010, with a final 2-year follow-up in 2012. We used audio computer-assisted self-interviewing [22] for all self-reports except for information on conduct problems which was collected via paper and pencil forms. Most of the samples (70%) were students at 7 public and private schools in the Philadelphia area. The remaining 30% of participants were recruited via flyers distributed at other local venues (e.g., libraries) [21]. We received assent from the adolescents and informed consent from their parents.

The present study only uses waves 3 to 6 due to low levels of alcohol use in prior waves. These 4 waves will be referred to as T1 (baseline), T2, T3, and T4. Attrition was 5.3%, 5.7%, 13.4%, and 24.8% from waves 3 to 6, respectively. Valid data were available for 364 adolescents (at baseline: 51.8% female; M_age_ = 13.51 years). At T4, participants were between ages 18–21 (*M*_age_ = 18.78 [0.72]). We define T1 as early adolescence, T2 and T3 as mid adolescence, and T4 as late adolescence (or “emerging adulthood”). The participants were diverse in ethnicity: 55.07% non-Hispanic White, 26.85% non-Hispanic Black, 9.04% Hispanic, and 9.04% identified as other [10,21]. Most participants had a low-middle (socio-economic status [SES]; *Hollingshead Two-Factor Index of Social Status*: M = 47.38 ± 15.43; reversed scored) [10].

### Measures

*Impulsivity* (T1eT4) was measured using 13 yes/no items (e.g., ‘Do you usually do and say things without stopping to think?’) from the Junior Eysenck Impulsivity Scale [23,24], which has been validated in previous studies (e.g., [25]). We focused on the acting without thinking about dimension of impulsivity, which pertains to behavior undertaken without adequate consideration of its consequences [26] because (1) it has been consistently linked to both substance use and antisocial behavior [18–20]; and (2) it is theoretically related to the behavioral disinhibition concept of BDM [14]. We summed the scores on these items, and thus this resulting sum-score had a continuous scale, ranging from 0 to 13. Higher scores indicated more impulsive behavior. Cronbach alpha’s across waves 1–4: 0.78, 0.82, 0.77, and 0.80.

*Alcohol use* (T1–T4) was measured with two items that measured ever (yes/no) and past 30-day use. These two items were combined and recoded into one scale, with the following response categories: never drank (= 0), used but not in past 30 days (= 1), drank 1–9 days in past 30 days (=2), and drank 10–30 days (= 3).

AUD criteria (AUDc) at T4 were measured with a questionnaire that tapped abuse and dependence as defined in the Diagnostic and Statistical Manual of Mental Disorders (DSM-IV) [27,28]. We used these items to measure AUD as per DSM-5 criteria which do not distinguish between abuse and dependence, but our measure did not assess craving. The scale consisted of 12 items and the following categories were used: 0 = 0 criteria met, 1 = 1 criterion met, 2 = 2 criteria met, and 3 = 3 criteria met. The final 2 categories correspond to a mild AUD diagnosis as per DSM-5 [24]. A total of 13.5% of participants met criteria for a mild AUD. Nevertheless, we used the criterion scores (range: 0e3) to measure severity of AUDc.

*Antisocial behavior* was assessed using 15 items of the Conduct Problems scale of the Youth Self Report [29,30] at T1–T3 that assess symptoms of conduct disorder as defined in DSM-IV. This scale measures antisocial behaviors such as “stealing” and “physically attacking people”, within the last 6 months, on a scale of: nottrue (=0), somewhator sometimes true (=1), and very true or often true (=2). There were no items pertaining to substance use. At T4, participants were 18 years or older; thus the Antisocial Personality Problems scale on the Adult Self-Report [30] was used to assess antisocial behaviors. This scale consists of 20 items that tap the symptoms of APD as defined in DSM-IV, with no items pertaining to substance use. Reliabilities for these scales [31] are high (Cronbach alpha’s >0.70) and they have been validated in other studies [31]. Mean scores were used for both scales.

*APDc* were measured at T4 using DSM-IV criteria [30,32]. This DSM-oriented scale comprises 20 items that were rated as being very consistent with APD [32]. The following categories were used: 1 = normal; 2 = borderline; and 3 = clinical. A total of 5.2% adolescents met the borderline range and 2.4% met the clinical range.

### Strategy of analyses

The RI-CLPM was specified in *Mplus* 7.3 [33]. This model included stability paths for antisocial behavior, alcohol use, impulsivity, and cross-lagged paths between these variables. Concurrent associations between these variables of interest were also estimated in this model. Furthermore, we controlled for gender, race, ethnicity, and SES. Next, the variance between persons (i.e., stable time invariant traits) was parceled out from the variance of the observed scores [34], by controlling the random intercept in the scores of antisocial behavior, substance use, and impulsivity. As a result, the cross-lagged linkages signify whether a within-person change in alcohol use, for example, can be predicted by deviation from one’s own mean score on impulsivity and vice versa [34]. We constrained the cross-lagged paths to be equal [34] across T1–T3 as the time-lag between these time points were equal and since this did not worsen the model fit, *X*^2^(6) = 8.90, p = .179. To account for developmental changes in the means, we did not constrain the means to be equal over time [35]. Finally, to test for indirect effects between initial impulsivity and T4 AUD and APD, we included cross-sectional paths between T4 alcohol use and antisocial behavior with those outcomes.

To test the significance of the mediational paths, we conducted mediation analyses using bootstrapped standard errors [36]. Given no apparent bias in participant dropout [17,37], we used Full Information Maximum Likelihood for missing data [33]. The magnitude of these cross-lagged paths were interpreted as 0.03 (small effect), 0.07 (medium effect), and 0.12 (large effect) [38].

## Results

The means of alcohol use increased over the waves but antisocial behavior and impulsivity peaked at T3 and T2, respectively, and declined thereafter (Table 1). All variables were concurrently correlated in the expected directions at each wave (Table 2).

### Random intercept cross-lagged panel model

**Figure 2.**
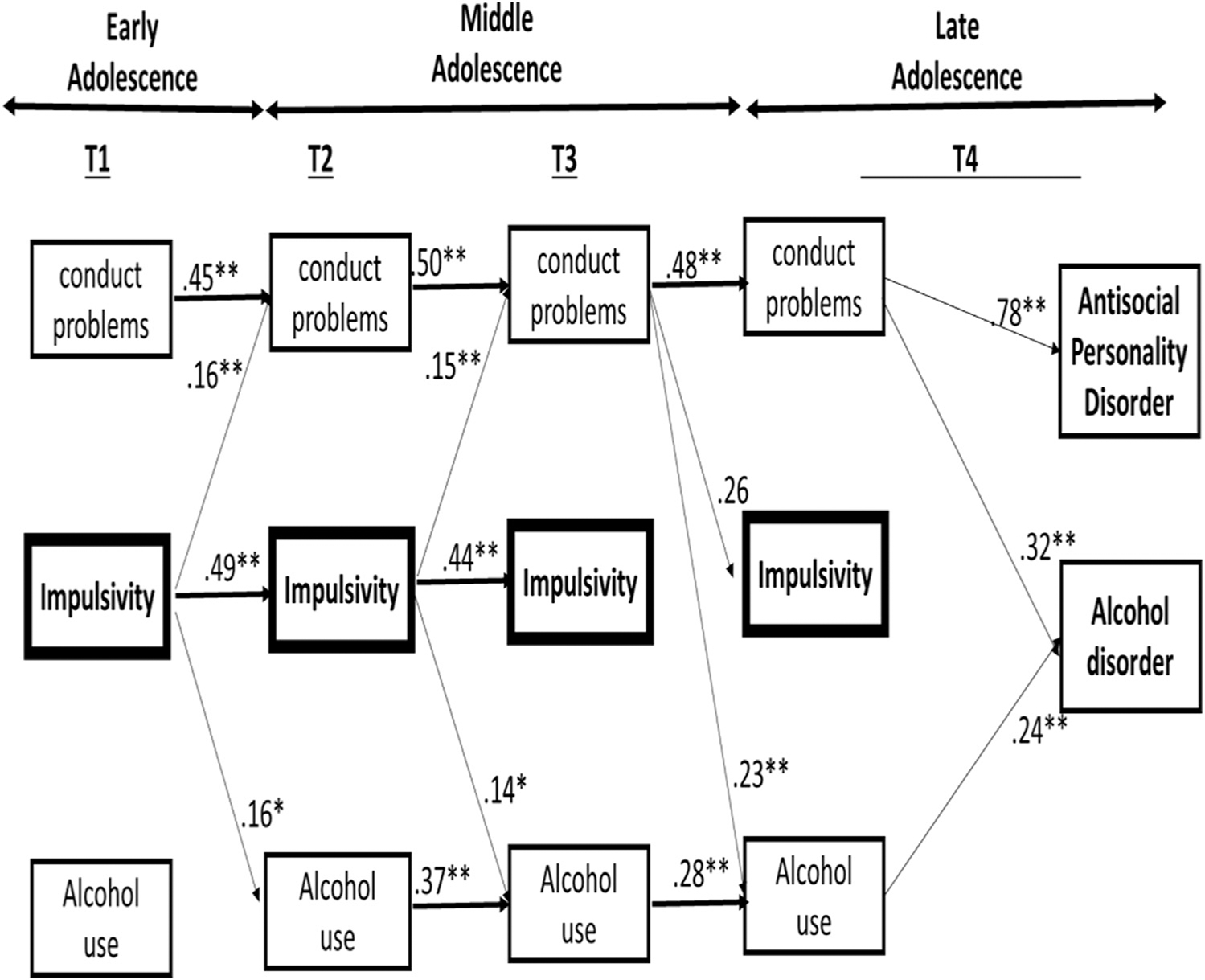
Significant standardized paths of the final RI-CLPM. Within-wave associations and control variables (gender, SES, and ethnicity) are not depicted.

The results of the final model are reported in Figure 2 and Tables 3 and 4. While the cross-sectional path between T4 impulsivity and T4 APDc was not significant (β = .043; *p* = .506), the cross-sectional path between T4 impulsivity and T4 AUDc was significant (β = .235; *p* = .004). But we found that (1) there was no stability in impulsivity from T3 to T4; (2) no direct cross-lagged paths from T3 impulsivity to T4 alcohol use or from T3 impulsivity to T4 antisocial behavior; and (3) no significant indirect mediational effects via T4 impulsivity to T4 AUDs or from T4 impulsivity to T4 APDc. Therefore, we constrained the paths between T4 impulsivity and T4 AUD and APD to be zero. The fit of the model (Chi-squared (57) = 113.603 *p* < .001) including these constraints was good (Comparitive Fit Index = 0.98; Root Mean Square Error of Approximation = 0.05; Standardized Root Mean Squared Residual = 0.04). The Bayesian Information Criterion also confirmed that the more parsimonious model without those paths provided a good fit (Bayesian Information Criterion = 9255.241 vs. 9255.414). These constraints are consistent with BDM, which posits that early adolescent impulsivity (T1) predicts APD and AUD (T4) via mid-adolescent (T2-T3) antisocial behavior and alcohol use (and not via impulsivity). Nevertheless, as a robustness check, we also tested whether impulsivity at T3 might predict subsequent T4 AUD and APD. However, this link and any indirect paths via this link were nonsignificant.

Taken together, results showed that changes in impulsivity only predicted changes in alcohol use and antisocial behavior in the first two waves of the series. In other words, impulsivity was no longer a significant predictor from T3 to T4. Instead, T3 antisocial behavior predicted both T4 alcohol use and T4 impulsivity. As for indirect effects, significant mediational cascading links from T1 impulsivity (via antisocial behavior) to T4 AUD (Total indirect: B = 0.014, 95% CI = 0.003, 0.026, *p* = .017; *β* = 0.043, 95% CI = 0.008, 0.082, *p* = .023) and to T4 APD (Total indirect: B = 0.009, 95% CI = 0.001, 0.016, *p* = .019; *β* = 0.062, 95% CI = 0.009, 0.104, *p* = .015) emerged. The link from T1 alcohol use to T2 alcohol use was nonsignificant, and thus we tested the mediational path beginning at T2 alcohol use instead of T1. Not surprisingly, we found significant mediational paths from T2 alcohol use to T4 AUDc (Total indirect: B = 0.050; 95% CI = 0.007, 0.106, *p* = .046; β = 0.039, 95% CI = 0.005, 0.084; *p* = .055) and significant mediational paths from T1 antisocial behavior to T4 APD (Total indirect: B = 0.173; 95% CI = 0.057, 0.345, *p* =. 017; *β* = 0.091; 95% CI = 0.034, .162, *p* = .015). For the specific indirect effects, please refer to Table 4. The significant cross-lagged paths were large in magnitude [38], while the significant indirect paths were small in magnitude. To verify our decision to only focus on one form of impulsivity, we also tested a model using delay discounting as the indicator of impulsivity but did not observe any indirect effects of this form of impulsivity to late adolescent psychopathology via mid-adolescent problem behaviors.

## Discussion

Guided by the BDM [1], we investigated whether cascading mediating links exist from early adolescent impulsivity to late adolescent/emerging adulthood AUD and/or APD via mid-adolescent alcohol use and/or antisocial behavior (problem behaviors). Consistent with the BDM, the results showed that from early to mid-adolescence changes in impulsivity predicted changes in antisocial behavior and alcohol use. However, changes in impulsivity did not predict changes in antisocial behavior and alcohol use from mid-adolescence to late-adolescence. Similarly, other studies suggest that adolescent impulsivity is less predictive of problem behaviors after mid-adolescence or late-adolescence [12–15]. Unlike those studies, the present study included repeated assessments of impulsivity (and problem behaviors), spanning early to late adolescence, and thus more reliably determines whether the predictive power of impulsivity for problem behaviors is the same during the different stages of adolescence.

Our findings appear to be inconsistent with a previous study [39] that found that declines in impulsivity were correlated with declines in problematic alcohol use in adulthood. Of note, unlike this study, the growth models used in that study [39] did not disentangle a possible role of antisocial behavior, which limits the comparisons between the results of that study with the present study. Nevertheless, similar to that study [39], we also found correlations between impulsivity and alcohol use in our adolescent sample, although impulsivity did not consistently predict the changes in those outcomes as adolescents aged.

Of note, we found that whereas impulsivity predicted problem behavior (and not vice versa) from early to mid-adolescence, a reverse link emerged in mid-adolescence to late-adolescence with increases in antisocial behavior predicting increases in impulsivity. The BDM does not hypothesize such a reversed link. Nevertheless, at least four studies [40–43] that investigated this reversed link have found evidence for it. Perhaps, individuals who engage in antisocial behavior may be labeled as “rule breakers” [18,41–43], which could lead them to describe themselves as more impulsive as they age. This interpretation, extrapolated from labeling theory within the criminology–sociology literature, suggests that labeling someone as criminal or antisocial causes the individual to assume similar attributes [44]. Adolescents might be even more susceptible to such labeling effects due to their ongoing identity development. It would be of an added value for future research to explore this hypothesis.

As for mediational links, we further found support for the BDM, as mediational analyses showed that early adolescent impulsivity predicted late-adolescent APD via mid-adolescent antisocial behavior. However, we did not find that early adolescent impulsivity predicted late-adolescent AUD via mid-adolescent substance use. Instead, we found that early adolescent impulsivity predicted late-adolescent AUD via mid-adolescent antisocial behavior. These mediational results showed small mediation effects but they are nevertheless consistent with the conclusion that impulsivity is a factor in early development of adolescent problem behavior but that this influence wanes as youth develop such that its effects are only evident in the consequences of early impulsivity on alcohol use and antisocial behavior.

To the best of our knowledge, this is the first study to investigate cascading mediating links between impulsivity, alcohol use, and antisocial behavior across adolescence. Nevertheless, at least one study similarly showed that both early adolescent hyperactivity/impulsivity and antisocial behavior predicted alcohol use by age 14 [16]. In addition, early hyperactivity/impulsivity predicted AUD [16]. Our results are consistent with those results [16] and further show that the link between early impulsivity and late AUD is mediated by intervening changes in other problem behavior, beyond within-wave associations and controls for ethnicity, gender, and SES.

In sum, in a large, ethnically, and socioeconomically diverse community sample, we found evidence of antisocial behavior not only as a consequence of impulsivity but also as a predictor of alcohol use from mid-adolescence to late-adolescence. In other words, mid-adolescent antisocial behavior (T3) continued to predict substance use 2 years later in late adolescence/emerging adulthood (T4), whereas mid-adolescent impulsivity at T3 did not. Zooming in on the sequencing of antisocial behavior and alcohol use, we only found evidence of a unidirectional link from antisocial behavior to alcohol use [45]. Finally, as mentioned previously, antisocial behavior additionally predicted AUD, whereas impulsivity did not. Taken together, these results imply that antisocial behavior is more relevant than impulsivity in predicting alcohol use, AUD, and APD in late adolescence. However, intervening early is critical to further avoid the consequences of impulsivity which are more difficult to reverse once psychopathology has developed. Prevention of heightened impulsivity in earlier years also needs attention, in particular focusing on the childhood precursors of impulsivity during early adolescence. Research suggests that individual factors such as childhood executive functioning and social factors such as parenting styles during childhood are important to consider [46]. In the present study, SES effects were accounted for and we found that SES was a significant predictor of impulsivity at each wave. Future research could further investigate the mechanisms by which early exposure to socioeconomic disadvantage influences heightened impulsivity during adolescence, including impacts on child executive functioning and parenting behaviors [47,48]. As for prevention and intervention programs, individual-level interventions such as mindfulness training have shown promising effects for reducing both impulsivity [49] and antisocial behavior [50] in youth. Family-based interventions have also shown promising results [51].

### Limitations and future directions

Despite the novel aspects of this study, some caveats should be considered when interpreting the results. Inspired by the BDM, the present study focused on impulsivity as an individual-level predictor. Although social risk factors (e.g., deviant friends) are also acknowledged by this model, impulsivity is hypothesized to be the common risk factor that leads to such social risk factors. Nevertheless, social influences should also be considered in future tests of the model. Of note, a prior study [17] using the current sample showed via latent growth modeling that even the subgroup trajectory wherein impulsivity increased in early adolescence and declined thereafter, still predicted late SUD in late adolescence. The present study further suggests that in that trajectory group, increased levels of antisocial behaviors (as a result of increased impulsivity in early adolescence) predicted the SUD that was observed in that study. Hence, future studies are encouraged to consider antisocial behavior (in addition to impulsivity) when investigating SUD. As for measurement limitations, of note is that our impulsivity measure used binary (yes/no) response options, which may not have adequately captured the variability in impulsivity, as compared to a continuous measure. Nevertheless, research suggests that binary response formats versus ordinal multicategory response formats are equally reliable [52]. Finally, our results for AUD and APD were based on questionnaire responses that have been validated in past research but may not be as robust as diagnoses determined by trained interviewers.

### Conclusion

Current results show that targeting impulsivity early in adolescence could halt the cascading chain of events leading to late adolescent AUD and APD, by slowing growth in antisocial behavior during mid-adolescence. However, it appears that during mid to late adolescence, intervening on the consequences of impulsivity (i.e., antisocial behavior) might be more useful to treat alcohol use, AUD, and APD rather than intervening on impulsivity itself. Thus, the findings suggest the importance of antisocial behavior as a source of risk for alcohol use, AUD, and APD in late adolescence. The prevalence rates of AUD and APD in the current ethnically and socioeconomically diverse sample were comparable to nationally representative samples [53,54], and hence the current results may apply to community-based samples of youth. To conclude, clinicians should be aware that our results suggest that adolescents with increasing levels of antisocial behavior are at risk of both AUD and APD.

## Supplementary Material

Supplement to Defoe et al., 2022

## Figures and Tables

**Figure 1.**
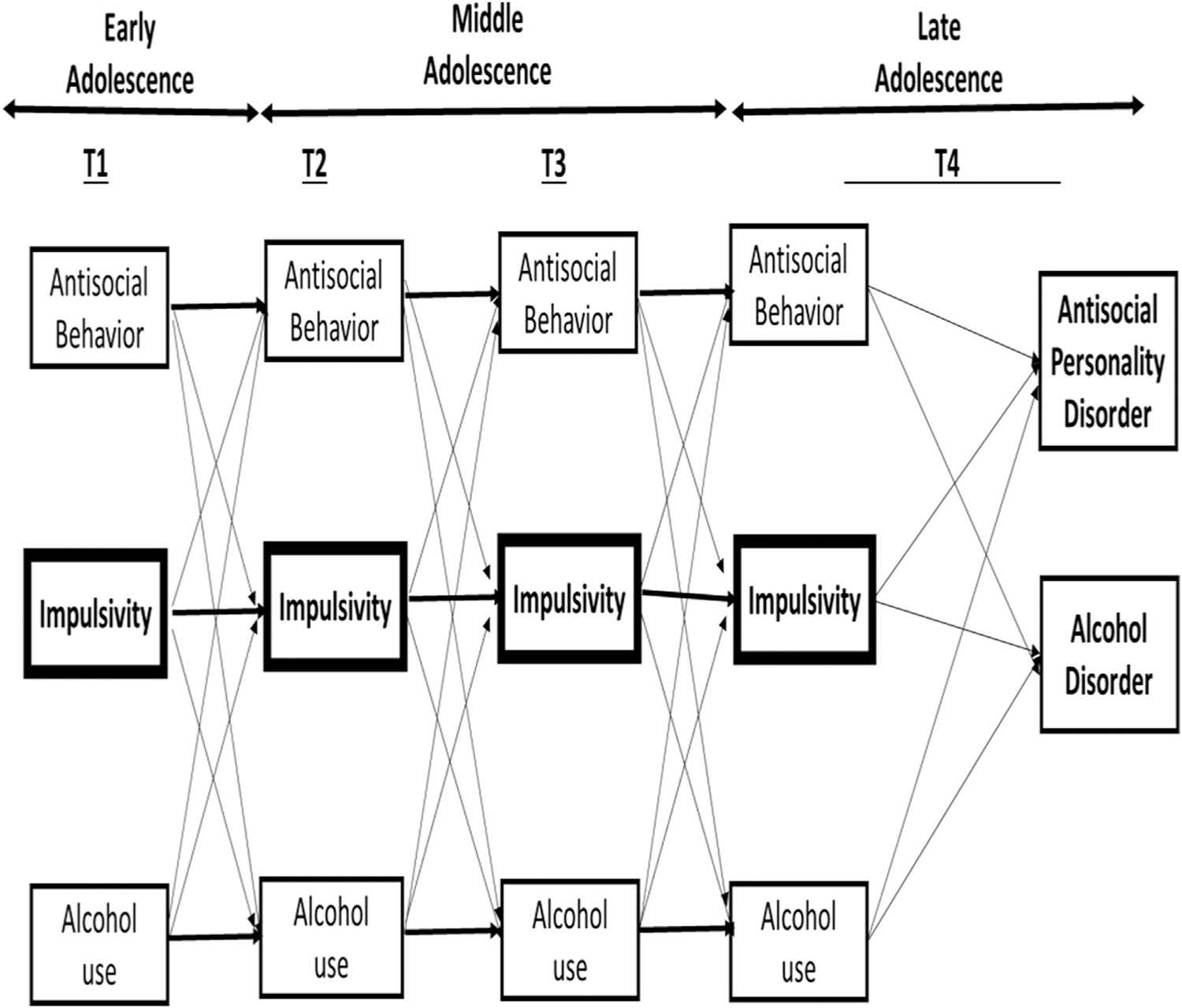
All examined paths of the RI-CLPM. Within-wave associations and control variables (gender, SES, and ethnicity) are not depicted. In the final RI-CLPM, all paths from T4 impulsivity to T4 Alcohol Use Disorder symptoms and from T4 impulsivity to T4 Antisocial Personality Disorder symptoms could be constrained to 0.

**Table 1:** Descriptive statistics of variables in the RI-CLPM model

Variables	Minimum	Maximum	Mean	SD

T1 Alcohol use	0	3.00	0.44	0.70
T2 Alcohol use	0	3.00	0.53	0.76
T3 Alcohol use	0	3.00	0.77	0.89
T4 Alcohol use	0	3.00	1.42	0.87
T4 AUDc	0	3.00	0.47	0.85
T1 Impulsivity	0	13.00	5.19	3.29
T2 Impulsivity	0	13.00	5.23	3.53
T3 Impulsivity	0	12.00	4.76	3.20
T4 Impulsivity	0	13.00	4.07	3.24
T1 Antisocial behavior	0	1.40	0.22	0.23
T2 Antisocial behavior	0	1.00	0.24	0.21
T3 Antisocial behavior	0	1.27	0.27	0.24
T4 Antisocial behavior	0	1.00	0.22	0.19
T4 APDc	1	3.00	1.10	0.37

APDc = Antisocial Personality Disorder criteria; AUDc = Alcohol Use Disorder criteria.

**Table 2:** Correlations between variables in the RI-CLPM model

Variables	1.	2.	3.	4.	5.	6.	7.	8.	9.	10.	11.	12.	13.	14.

1.T1 AU	-													
2. T2 AU	0.53**	-												
3. T3 AU	0.47**	0.56**	-											
4. T4 AU	0.32**	0.34**	0.47**	-										
5. T4 AUD	0.18**	0.19**	0.22**	0.34**	-									
6. T1 AB	0.46**	0.35**	0.30**	0.17**	0.15*	-								
7. T2 AB	0.38**	0.46**	0.36**	0.19**	0.13*	0.68**	-							
8. T3 AB	0.30**	0.31**	0.42**	0.29**	0.21**	0.64**	0.69**	-						
9. T4 AB	0.19**	0.26**	0.25**	0.29**	0.32**	0.40**	0.43**	0.57**	-					
10. T4 APD	0.09	0.15*	0.10	0.16**	0.39**	0.21**	0.21**	0.33**	0.68**	-				
11. T1 IMP	0.36**	0.30**	0.31**	0.22**	0.10	0.56**	0.48**	0.50**	0.34**	0.14*	-			
12. T2 IMP	0.32**	0.33**	0.32**	0.18**	0.14*	0.49**	0.55**	0.47**	0.29**	0.09	0.71**	-		
13. T3 IMP	0.09	0.14**	0.26**	0.13*	0.12*	0.33**	0.36**	0.49**	0.34**	0.17**	0.54**	0.68**	-	
14. T4 IMP	0.14*	0.17**	0.15*	0.15**	0.29**	0.29**	0.29**	0.35**	0.48**	0.30**	0.44**	0.52**	0.53**	-

* *p* < .05;

** *p* < .01

AB = Antisocial Behavior; AU = Alcohol Use; IMP = impulsivity.

**Table 3:** RI-CLPM: Links between alcohol use, impulsivity, antisocial behavior, alcohol use disorder criteria, and antisocial personality disorder criteria

Parameters	B	SE (B)	β	SE (β)

Cross-lagged paths				
T1 Impulsivity → T2 Antisocial	0.01**	0.00	0.16**	0.06
T1 Alcohol → T2 Antisocial	0.02	0.02	0.07	0.05
T2 Impulsivity → T3 Antisocial	0.01**	0.00	0.15**	0.05
T2 Alcohol → T3 Antisocial	0.02	0.02	0.07	0.05
T3 Impulsivity → T4 Antisocial	0.00	0.01	0.02	0.09
T3 Alcohol → T4 Antisocial	0.00	0.02	0.01	0.09
T1 Impulsivity → T2 Alcohol	0.04*	0.02	0.16*	0.07
T1 Antisocial → T2 Alcohol	0.41	0.25	0.12	0.07
T2 Impulsivity → T3 Alcohol	0.04*	0.02	0.14*	0.07
T2 Antisocial → T3 Alcohol	0.41	0.25	0.10	0.06
T3 Impulsivity → T4 Alcohol	−0.01	0.02	−0.04	0.08
T3 Antisocial → T4 Alcohol	0.82**	0.28	0.23**	0.04
T1 Alcohol → T2 Impulsivity	0.12	0.28	0.03	0.06
T1 Antisocial → T2 Impulsivity	1.66	0.96	0.12	0.07
T2 Alcohol → T3 Impulsivity	0.12	0.28	0.03	0.07
T2 Antisocial → T3 Impulsivity	1.66	0.96	0.12	0.07
T3 Alcohol → T4 Impulsivity	−0.25	0.36	−0.08	0.11
T3 Antisocial → T4 Impulsivity	3.12*	1.28	0.26*	0.11
Final links to T4 AUDc				
T4 Antisocial → T4 AUDc	1.64**	0.46	0.32*	0.09
T4 Alcohol → T4 AUDc	0.27**	0.06	0.24*	0.05
Final links to T4 APDc				
T4 Antisocial → T4 APDc	1.77**	0.26	0.78*	0.06
T4 Alcohol → T4 APDc	−0.03	0.03	−0.05	0.05
T1 Correlations				
T1 Antisocial - T1 Alcohol	0.06**	0.01	0.49**	0.08
T1 Antisocial - T1 Impulsivity	0.28**	0.06	0.53**	0.06
T1 Alcohol - T1 Impulsivity	0.61**	0.18	0.39**	0.09
Correlated residuals				
T2 Antisocial - T2 Alcohol	0.03**	0.01	0.32**	0.06
T2 Antisocial - T2 Impulsivity	0.11**	0.03	0.33**	0.07
T2 Alcohol - T2 Impulsivity	0.18	0.10	0.13	0.08
T3 Antisocial - T3 Alcohol	0.03**	0.01	0.29**	0.07
T3 Antisocial - T3 Impulsivity	0.13**	0.03	0.35**	0.07
T3 Alcohol - T3 Impulsivity	0.21*	0.10	0.14	0.07
T4 Antisocial - T4 Alcohol	0.02*	0.01	0.15*	0.07
T4 Antisocial - T4 Impulsivity	0.14**	0.04	0.39**	0.08
T4 Alcohol - T4 Impulsivity	0.14	0.13	0.08	0.08

* *p* < .05;

** *p* < .01

SE = standard error; AUDc = Alcohol Use Disorder criteria; APDc = Antisocial Personality Disorder criteria.

**Table 4:** Significant specific mediational links to alcohol use disorder criteria (AUDc) and antisocial personality disorder criteria (APDc)

Parameters	B	95% CI

Mediational links from T2 alcohol use to T4 AUDc		
T2 AU→T3 AU→T4 AU→T4 AUDc	0.032	0.008, 0.070
Mediational links from T1 Antisocial behavior to T4 APDc		
T1 AB→T2 AB→T3 AB→T4 AB→T4 APDc	0.159	0.049, 0.321
Mediational links from T1 Impulsivity to T4 AUDc		
T1 IMP- > T2 AB- > T3 AB- > T4 AB- > T4	0.004	0.001, 0.008
AUDc T1 IMP- > T2 IMP- > T3 AB- > T4 AB- > T4 AUDc	0.004	0.001, 0.007
Mediational links from T1 Impulsivity to T4 APDc		
T1 IMP- > T2 AB- > T3 AB- > T4 AB- > T4 APDc	0.004	0.001, 0.008
T1 IMP- > T2 IMP- > T3 AB- > T4 AB- > T4 APDc	0.004	0.001, 0.007

AB = Antisocial Behavior; APDc = Antisocial Personality Disorder criteria; AU = alcohol use; AUDc = Alcohol use Disorder criteria; IMP = Impulsivity.
