# Supplementary material for: Cascades From Early Adolescent Impulsivity to Late Adolescent Antisocial Personality Disorder and Alcohol Use Disorder
Source: J Adolesc Health. Author manuscript; Available in PMC 2024 Jun 18. (PMC11184504; doi:10.1016/j.jadohealth.2022.06.007)
Supplement: Supplement to Defoe et al., 2022 [file NIHMS1997658-supplement-Supplement_to_Defoe_et_al___2022.docx]

| **Paths** | β | SE(β) |
| --- | --- | --- |
| **T1 Antisocial behavior with:** |  |  |
| Gender | .17** | .05 |
| SES | .17** | .04 |
| Black | .20** | .06 |
| Hispanic | .01 | .05 |
| Other | -.08* | .04 |
|  |  |  |
| **T2 Antisocial behavior with:** |  |  |
| Gender | .22** | .05 |
| SES | .14** | .05 |
| Black | .18** | .05 |
| Hispanic | .08 | .07 |
| Other | -.07 | .04 |
|  |  |  |
| **T3 Antisocial behavior with:** |  |  |
| Gender | .18 | .06 |
| SES | .10 | .05 |
| Black | .18 | .05 |
| Hispanic | .04 | .07 |
| Other | -.07 | .04 |
|  |  |  |
| **T4 Antisocial behavior with:** |  |  |
| Gender | .11* | .05 |
| SES | .01 | .04 |
| Black | .15** | .05 |
| Hispanic | -.02 | .05 |
| Other | .01 | .04 |
|  |  |  |
| **T1 Alcohol with:** |  |  |
| Gender | .15** | .05 |
| SES | .02 | .05 |
| Black | .04 | .05 |
| Hispanic | .03 | .06 |
| Other | -.10** | .04 |
|  |  |  |
| **T2 Alcohol with:** |  |  |
| Gender | .11* | .05 |
| SES | .12* | .05 |
| Black | .02 | .06 |
| Hispanic | -.05 | .05 |
| Other | -.15** | .04 |
|  |  |  |
| **T3 Alcohol with:** |  |  |
| Gender | .07 | .06 |
| SES | .10 | .05 |
| Black | -.09 | .06 |
| Hispanic | .03 | .06 |
| Other | -.20** | .04 |
|  |  |  |
| **T4 Alcohol with:** |  |  |
| Gender | .05 | .05 |
| SES | -.15** | .06 |
| Black | -.04 | .05 |
| Hispanic | -.04 | .06 |
| Other | -.18** | .07 |
|  |  |  |
| **T1 Impulsivity with:** |  |  |
| Gender | .09 | .05 |
| SES | .14** | .05 |
| Black | .22** | .05 |
| Hispanic | .02 | .06 |
| Other | -.02 | .05 |
|  |  |  |
| **T2 Impulsivity with:** |  |  |
| Gender | .17** | .05 |
| SES | .16** | .05 |
| Black | .17** | .05 |
| Hispanic | .00 | .06 |
| Other | -.01 | .06 |
|  |  |  |
| **T3 Impulsivity with:** |  |  |
| Gender | .09 | .06 |
| SES | .14** | .05 |
| Black | .14* | .06 |
| Hispanic | .02 | .06 |
| Other | -.01 | .06 |
|  |  |  |
| **T4 Impulsivity with:** |  |  |
| Gender | .12* | .03 |
| SES | .18** | .00 |
| Black | .11 | .06 |
| Hispanic | -.07 | .20 |
| Other | -.01 | .89 |
